# Decrease in air-sea CO_2_ fluxes caused by persistent marine heatwaves

**DOI:** 10.1038/s41467-022-31983-0

**Published:** 2022-07-25

**Authors:** Alexandre Mignot, Karina von Schuckmann, Peter Landschützer, Florent Gasparin, Simon van Gennip, Coralie Perruche, Julien Lamouroux, Tristan Amm

**Affiliations:** 1 Mercator Océan International, Toulouse, France; 2 Max Planck Institute for Meteorology, Hamburg, Germany

**Keywords:** Marine chemistry, Physical oceanography

## Abstract

Regional processes play a key role in the global carbon budget. Major ocean CO_2_ uptake at mid-latitudes counteracts CO_2_ release in the tropics, which is modulated by episodes of marine heatwaves. Yet, we lack essential knowledge on persistent marine heatwaves, and their effect on the CO_2_ sensitive areas. Here we show, using a 1985–2017 joint analysis of reconstructions, ocean reanalysis and in situ and satellite data, that persistent marine heatwaves occur in major CO_2_ uptake and release areas. Average air-sea CO_2_ flux density changes from persistent marine heatwaves are strongest in the Pacific Ocean with a 40 ± 9% reduction in CO_2_ release in the tropics linked to ENSO, and a reduction in CO_2_ uptake of 29 ± 11% in the North Pacific over the study period. These results provide new insights into the interplay of extreme variability and a critical regulating ocean ecosystem service, and pave the way for future investigations on its evolution under climate change.

## Abstract (web summary)

Ocean CO_2_ uptake at mid-latitudes counteracts CO_2_ release in the tropics, but we know little about the effects of marine heatwaves that modulate this process. Here, the authors use joint analysis of satellite measurements, in situ observation, reconstructions derived from machine learning algorithms, numerical model of the global ocean, and find that areas where PMHWs most frequently occur coincide with the regions that are the most critical for the oceanic carbon cycle.

## Introduction

Extreme events associated with unusually high-water temperature are ubiquitous in the global ocean. They can last from weeks to years, span from local to interbasin scale, and can reach depths of several hundreds of meters^1–3^. These so-called marine heatwaves (MHWs) occur due to either coupled air-sea interactions^4–7^, ocean internal processes such as horizontal and/or vertical circulation changes^8^, and are sometimes linked to large climate modes such as the El Niño-Southern Oscillation (ENSO)^5^. Over the past 35 years, MHWs have become longer-lasting, more intense and more extensive^9–11^ very likely due to long-term anthropogenic change^1,9,10,12,13^.

Intense and long-lasting MHWs have been reported at different locations in the global ocean^3,5^. These include the 2013/2015 Northeast Pacific ‘warm blob’^4,14^, the 1997/1998 El Niño^15^, the 2015/2016 Tasman Sea^16^ or the 2012 Northwest Atlantic^17,18^. The duration of these major events ranges from several months up to 2 years, they are associated with dramatic increase in sea surface temperature, that can exceed 5 °C in anomalies^19^, and they can extend over large regions, reaching sometimes ~ 10 M km^2^ ^5^. Due to their extreme nature, MHWs, and in particular the intense, persistent ones, pose a fundamental challenge for societies as they have devastating impacts on marine ecosystem and their services^1,11,20^.

**Fig. 1:**
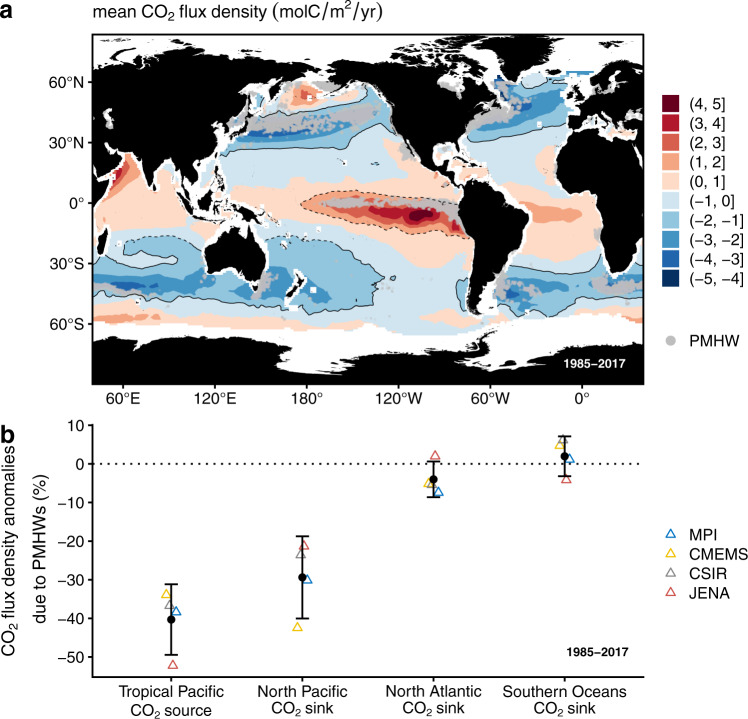
Interplay of PMHWs and oceanic carbon CO_2_ source and sink areas. **a** Mean 1985–2017 air-to-sea CO_2_ flux density (F_CO2_) derived from the Copernicus Marine Service (CMEMS) observation-based product (see methods section). Negative values indicate oceanic uptake (blue), while positive values indicate oceanic outgassing (red) of CO_2_. The black continuous/dashed contours represent critical CO_2_ sink/source regions, i.e the regions where the mean 1985–2017 F_CO2_ is lower/greater than −1/1 molC/m^2^/yr as proposed by Takahashi et al.^35^. The gray points represent satellite Sea Surface Temperature grid points that have experienced at least 3 PMHWs from 1985 to 2017 (see text for details). **b** Trimmed average percent F_CO2_ anomalies during PMHWs derived from an ensemble of four observation-based products of F_CO2_ (see section methods) in critical oceanic CO_2_ sinks and sources (plain and dashed contours in Fig. 1a) that are impacted by PMHWs. The percent F_CO2_ anomalies correspond to the monthly F_CO2_ anomalies divided by the monthly F_CO2_ climatological values (see section methods). Negative values corresponds to a reduction in both a source or a sink region. The 95% confidence interval for each trimmed average percent F_CO2_ anomalies are indicated in the Supplementary Table 1. We verified that all trimmed average percent F_CO2_ anomalies were significantly different from 0 using a Yuen’s trimmed mean test^59^. The ensemble mean and standard deviation are given in black. An additional 12% uncertainty resulting from uncertain gas exchange^60^ has been added to the ensemble spread. The calculation of the percent F_CO2_ anomalies is detailed in the method section.

The ocean acts as a net sink for atmospheric CO_2_ and is absorbing about a ¼ of CO_2_ anthropogenic emissions^21^ (2.6 ± 0.6 PgC/yr over the 2009–2018 period), thereby mitigating global warming. CO_2_ entering the ocean is then redistributed horizontally over large distances and into deep ocean layers where it is then stored for long time scales^22–24^. The magnitude and direction of the air-sea CO_2_ flux density (F_CO2_) vary widely in space and time, and depend on hydrographic conditions, the ocean circulation system, biological net production and air-sea interactions. As a result, major CO_2_ uptake areas are located at mid-latitudes, whereas CO_2_ release takes place predominantly in upwelling areas such as the tropical ocean^25–27^ (Fig. 1a).

Persistent MHWs linked to ENSO^5^ affect the Tropical Pacific CO_2_ source region and lead to significant reduction in CO_2_ outgassing^28–32^. However, we lack essential knowledge about how major MHWs events affect other oceanic CO_2_ sources and sink regions. Here we investigate the interplay and the impact of intense and long-lasting MHWs on the air-sea CO_2_ flux density at the global scale. The study is built on a combined use of reconstructions from 1985 to 2017, direct measurements, remote sensing data and an ocean reanalysis. We first present the regions where particularly intense and long-lasting MHWs most frequently occur. We then quantify the impact of these extreme ocean events on oceanic CO_2_ sink and sources areas. We further examine the interaction between these extreme ocean events and one critical oceanic CO_2_ sink region in the North Pacific Ocean. Finally, we discuss these results with existing knowledge on the mechanisms in the Tropical Pacific region to obtain a large-scale view of the prevailing mechanisms driving coupled changes between one of the regulating ocean ecosystem service and extreme variability.

## Results

### Persistent marine heatwaves occurrence and oceanic CO_2_ source and sink areas

In the Tropical Pacific, intense and long-lasting MHWs (hereinafter denoted persistent marine heatwaves, PMHWs) have a strong impact on air-sea CO_2_ fluxes^31^. We propose a specific new criteria to identify such PMHWs at the global scale based on the duration and the mean Sea Surface Temperature (SST) anomaly during a MHW. We first detect all MHWs that occurred from 1985 to 2017 by applying a standard MHW detection algorithm^2^ to NOAA gridded SST data derived from AVHRR sensor^33,34^ (see method section). The detection algorithm provides several metrics that describe MHWs, including the duration and the mean SST anomaly. Using these two metrics, we define PMHWs as MHWs whose duration and mean SST anomaly are greater than the 95th percentile of their global historical distribution, i.e., duration > 38 days and mean SST anomaly > 2.3 degrees Celsius. Finally, we focus on the regions where PMHWs have appeared several times over the past three decades, and that represent a recurring event effecting the ocean CO_2_ sink, similar to El Niño events in the Tropical Pacific. To do so, we only consider the points where PMHWs have re-occurred at least three times during the 1985–2017 period (gray points in Fig. 1a)–which correspond to 25% of all grid points that have experienced at least one PMHW.

PMHWs most frequently occur in the largest oceanic CO_2_ source and sink areas in the near-global ocean. The analysis is restricted to within 60°S and 60°N to exclude polar regions where periods of ice longer than 5 days obstruct the MHW detection algorithm (see method section). Critical sink regions are located at mid-latitudes in the Northern and Southern Hemispheres (solid contours in Fig. 1a), while ocean CO_2_ outgassing predominantly occurs in upwelling regions such as the Tropical Pacific (dashed contours in Fig. 1a) and correspond to regions where climatological F_CO2_ is lower/greater (sinks/sources) than −1/1 molC/m^2^/year (solid and dashed contours in Fig. 1a), as proposed by Takahashi et al.^35^. The range in longitude and latitude of the four ocean CO_2_ sink/source regions are indicated in Table 1. Note that the mid-high latitude Southern Oceans CO_2_ sink region includes the uptake area in the South Atlantic, Pacific, and Indian Oceans between 20.5° and 56.5°S, respectively. The climatological F_CO2_ values are illustrated for the 1985–2017 period using the observation-based Copernicus Marine Environment Monitoring Service (hereafter denoted CMEMS) product^36^ (see method section). Unexpectedly, we find that the regions with a strong occurrence of PMHWs (gray points in Fig. 1a) are mainly located in the strongest oceanic sources and sinks areas in the near-global ocean, particularly in the Pacific Ocean (Fig. 1a and Table 1). In the North Pacific and in the Tropical Pacific, the area impacted by PMHWs covers 3.8 and 2.9 * 10^6^ km^2^, respectively, which corresponds to 21% and 16% of the total area of these basins. Whereas, in the North Atlantic and in the mid-high latitude Southern Oceans, the area impacted by PMHWs covers only 10% and 5% of the total area of these basins.

**Table 1:** Ranges in longitude and latitude, total area, area and normalized area impacted by PMHWs of the four regions considered in this study

critical CO_2_ sink/source regions	Longitude range	Latitude range	Total Area (10^6^ km^2^)	Area impacted by PMHWs (10^6^ km^2^)	Area impacted by PMHWs/ total Area (%)
North Pacific CO_2_ sink	123.5°E-121.5°W	23.5°N–59.5°N	17.7	3.8	21
Tropical Pacific CO_2_ source	173.5°E-73.5°W	17.5°S–5.5°N	18.4	2.9	16
North Atlantic CO_2_ sink	74.5°W-7.5°E	30.5°N–65.5°N	11.0	1.1	10
Mid-high latitude Southern Oceans CO_2_ sink	179.5°W-122.5 W and 85.5°W-180°E	20.5°S–56.5°S	56	2.8	5

The PMHWs impact on F_CO2_ is quantified using an ensemble of four observation-based products of F_CO2_ from 1985 to 2017 (see method section). We use a common approach using the ensemble spread as first order uncertainty estimate^37^. We would like to note though, that regionally, the uncertainty might be larger resulting from the lack of direct measurements. Previous studies, however, show that the uncertainty as a result of data sparsity is in the order of the ensemble spread adopted here^38^. Additionally data products are limited by 1×1 degree (2.5×2 degree for the JENA product) resolution by design and we are unable to formally quantify the role of the resolution on the uncertainty of our study. Amongst the largest oceanic CO_2_ sinks and sources where PMHWs most frequently occur, the North Pacific and the Tropical Pacific are the most impacted. During PMHWs events, both areas suffer from a significant reduction in the air-sea CO_2_ flux density with a change in uptake (29 ± 11%) and outgassing (40 ± 9%) respectively. In contrast, in the North Atlantic and the mid-high latitude Southern Oceans CO_2_ sinks, the impact of PMHWs on F_CO2_ is close to 0 and negligible over the study period. We verified that, in these two regions, the small value is a general feature and is not just a cancellation of large positive and negative anomalies. The impact of PMHWs on F_CO2_ appears thus to be the most important in the Tropical and North Pacific, which is of considerable concern given their contributions to the global ocean carbon cycle^25–27^. The Tropical (dashed contour) and North Pacific (solid contour) represent a source and a sink of 0.52 and −0.60 PgC yr^−1^, respectively which corresponds to −36 and 42% of the annual near-global net flux while covering roughly 5% of the near-global ocean area.

### Persistent marine heatwaves and the North Pacific CO_2_ sink

We use a state-of-the-art ocean biogeochemical reanalysis^39^ (see supplementary information), validated against F_CO2_ reconstructions (Fig. S1) and in situ observations from Biogeochemical (BGC)-Argo floats^40,41^ (Fig. S2), to understand the interaction between PMHWs and F_CO2_ in the North Pacific from 2009 to 2017. Note that, the calculations relative to the 2009–2017 period are performed on those North Pacific regions where PMHWs have re-occurred several times since 1985, i.e. the SST grid points that have experienced at least 3 PMHWS from 1985 to 2017. The exchange of CO_2_ between the ocean and the atmosphere is driven by six variables^42^: wind, upper-ocean temperature, salinity, dissolved inorganic carbon (DIC) and alkalinity (ALK) as well as the atmospheric partial pressure of CO_2_. Temperature, salinity, DIC and ALK are influenced by hydrographic conditions, the ocean circulation system, and air-sea interactions. In addition, DIC and ALK are also influenced by the biological net production^42^. As observation-based products of F_CO2_ do not include these variables, the reanalysis becomes essential to pursue the analysis. The BGC reanalysis combines ocean circulation and biogeochemistry models together with in situ and satellite observations to provide a high degree of bio-physical realism^43^. The reanalysis skill is validated against the ensemble of observation-based products over the 2009–2017 period and in situ observations from an array of BGC-Argo floats during the 2013/2015 ‘warm blob’ PMHW (see supplementary Information). The reanalysis shows good agreement with the observation-based products in estimating F_CO2_ anomalies due to PMHWs in the North Pacific. The reanalysis also agrees well with the float observations in reproducing anomalies in the four oceanic drivers known to control F_CO2_ (temperature, salinity, DIC and ALK) during the ‘warm blob’.

**Fig. 2:**
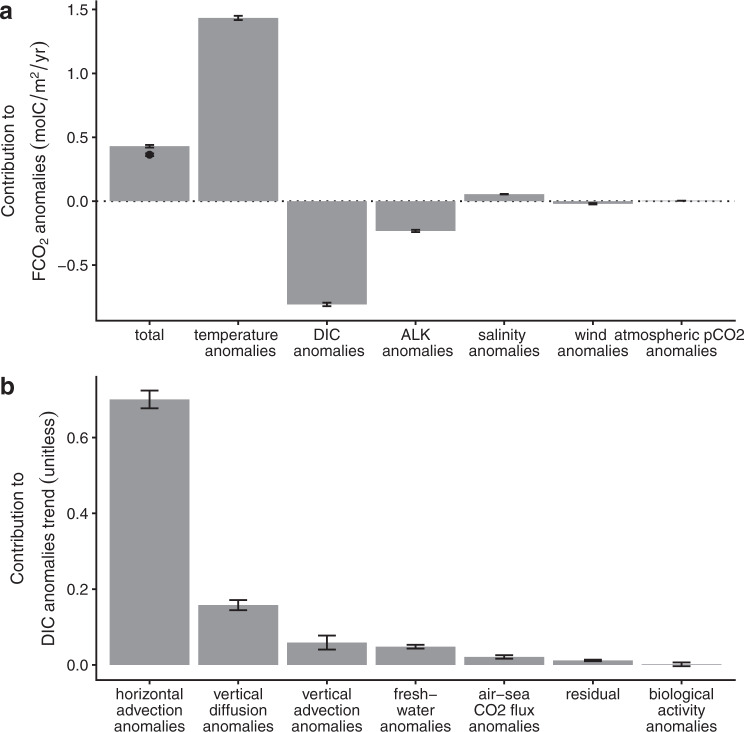
Processes that lead to a reduction in the oceanic uptake of CO_2_ in the North Pacific during PMHWs. **a** 2009–2017 average F_CO2_ anomalies (black dot) and its Taylor decomposition (vertical bars). The contribution of temperature, Dissolved Inorganic Carbon (DIC), Alkalinity (ALK), salinity, wind and atmospheric partial pressure of CO_2_ to F_CO2_ anomalies observed during PMHWs in the North Pacific CO_2_ sink region for the 2009–2017 period were calculated using a first order Taylor expansion derived from the biogeochemical reanalysis (see methods section). The “total” bar corresponds to the sum of all contributing terms and corresponds to the Taylor approximation of F_CO2_ anomalies (black dot). The good agreement between the two implies that F_CO2_ anomalies are well approximated by the Taylor decomposition. The error bars correspond to the 95% confidence interval. **b** Contribution of horizontal, vertical diffusion, vertical advection, dilution and concentration due to freshwater fluxes, air-sea CO_2_ flux density, a residual term and biological activity to the rate of change (tendency or trend) of DIC anomalies during PMHWs in the North Pacific CO_2_ sink region for the 2009–2017 period (see methods section). The vertical bars represent the slope from linearly regressing each forcing term to the DIC anomalies trend^28^. A linear regression slope close to 1 indicates that a particular term produces in-phase anomalies of comparable magnitude. A slope near zero indicates that the term is not important in generating anomalies. The error bars correspond to 95% confidence intervals.

During PMHW events, the reduction in F_CO2_ is the result of higher-than-usual temperature and negative DIC anomalies. We calculate a first-order Taylor series expansion of F_CO2_ anomalies to determine the contribution of the four oceanic drivers^28,44,45^ (see method section). The Taylor decomposition (Fig. 2a) reveals that the reduced uptake of CO_2_ during PMHWs in the North Pacific mainly result from the contribution of temperature (1.43 ± 0.02 molC/m^2^/yr), DIC anomalies (−0.81 ± 0.01 molC/m^2^/yr) and to a lesser extent ALK anomalies (−0.23 ± 0.01 molC/m^2^/yr). The contribution from salinity, wind and the atmospheric partial pressure of CO_2_ anomalies are small, and can be considered negligible. Sea surface warming during PMHWs reduces the solubility of CO_2_ in the ocean resulting in a reduced uptake of CO_2_. In contrast, the decrease in DIC associated with a small increase in ALK (Fig. S3) enhances the uptake of CO_2_ and as such offsets the thermal effect to the extent that the final F_CO2_ anomaly is ~4 times smaller than it would have been from the thermal effect alone.

Next, we investigate the mechanisms leading to negative DIC anomalies. We examine the processes that drive the rate of change (tendency or trend) of DIC anomalies during PMHWs over the 2009–2017 period. The budget (or forcing) terms in the DIC trend equation consist of: horizontal and vertical advection, vertical diffusion, the air-sea CO_2_ flux density, biological activity, dilution and concentration due to freshwater fluxes and a residual term (see method section). To highlight the contribution from each process to the DIC anomalies trend, we follow the method of Doney et al.^28^ and examine the slope through linear regression of each forcing term to the DIC anomalies trends (Fig. 2b) (we verify that the intercept is approximately 0 because the average of the forcing term anomalies is null). A slope close to 1 indicates that a particular forcing term produces in-phase anomalies of comparable magnitude to DIC anomalies trend. In contrast, a slope near zero indicates that the term is not important, and a negative slope that the term produces out of phase anomalies.

Horizontal advection is the main driver for DIC anomalies. The linear regression slope of horizontal advection on DIC anomalies trends is the largest (0.70 ± 0.02 (unitless)) whereas the slopes of the other forcing terms are much smaller (<0.16 (unitless) for vertical diffusion anomalies and lower than 0.06 (unitless) for all the other terms). Furthermore and consistently with Ayers and Lozier^46^ and Gruber et al.^23^, the reanalysis shows that, on average, there is a net horizontal divergence of DIC in the North Pacific CO_2_ sink region (data not shown). The reanalysis suggests that the lateral removal of DIC is further accentuated during PMHWs, causing a decrease in DIC. In the North Pacific, extreme MHWs are associated with changes in horizontal advection due to wind speed modification^4,19^. This suggests that similar processes could potentially both drive thermal and DIC changes during PMHWs in the North Pacific. Given the importance of the horizontal transport of DIC in mitigating the impact of PMHWs on the uptake of CO_2_, we propose that studies should address how PMHWs and ocean circulation are interconnected in this region.

## Discussion

**Fig. 3:**
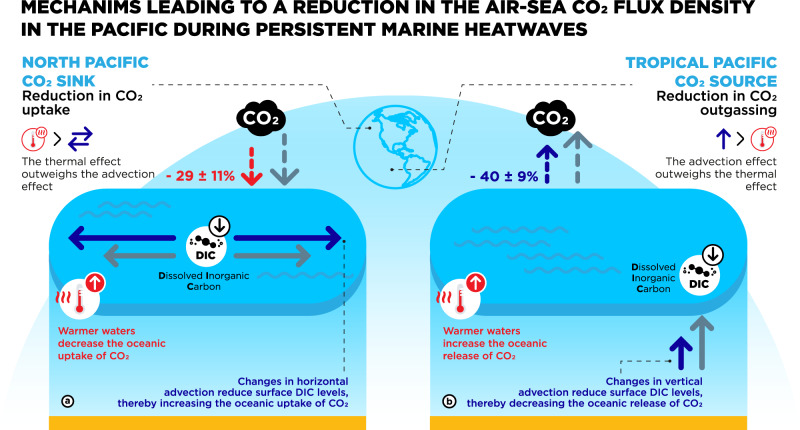
Schematic presentation of the mechanisms driving the reduction in the air-sea CO_2_ flux density in (a) the North Pacific CO_2_ sink and (b) the Tropical Pacific CO_2_ source regions. Red color indicates the thermal effect on the air-sea CO_2_ flux density, the blue color is linked to impacts related to circulations changes associated with PMHWs such as anomalous horizontal and vertical advection. The gray color represents the normal conditions. See text for more details.

We show that PMHWs (> 38 days and > 2.3 °C anomalies as defined in this study) most frequently occur in oceanic regions of major importance for the global carbon cycle: the Tropical Pacific CO_2_ source area, and the CO_2_ sink regions of the North Pacific, the North Atlantic and the mid-high latitude Southern Ocean. However, over the study period 1985–2017, PMHWs have impacted air-sea CO_2_ exchange only in the North and tropical Pacific CO_2_ sensitive areas. The processes of this interplay are provided in the schematic of Fig. 3.

In the North Pacific CO_2_ sink, PMHW events cause a reduction in the CO_2_ sink as a result of the net effect of two competing mechanisms: extreme higher-than-average temperature and anomalous DIC advection. The former causes a reduction in the solubility of CO_2_ in ocean water, thereby reducing the ocean uptake of CO_2_ whereas the latter increases the ocean CO_2_ uptake—through decreased levels of DIC driven by horizontal advection—and as such attenuates the impact of the thermal effect. Overall, the thermal effect dominates the advection effect in our study, leading to a net reduction in the air-to-sea CO_2_ flux density during a PMHW event of about 29 ± 11% (Fig. 3a).

In the Tropical Pacific where CO_2_ release takes place, the CO_2_ outgassing is significantly attenuated during PMHWs with a reduction in CO_2_ release from the ocean to the atmosphere of about 40 ± 9%. In this region, PMHWs are associated with ENSO^5^ and previous studies have investigated the mechanisms explaining this change, which is mainly driven by a change in vertical ocean circulation^28–32^. During PMHWs (Fig. 3b), eastward propagating Kelvin waves that depress the thermocline in the east together with a concurrent weakening of easterly winds, and the extension of the western Pacific warm pool to the east, reduce the upwelling of DIC leading to a net decrease in the sea-to-air CO_2_ flux density. We have only considered the average sea-to-air CO_2_ flux density response to PMHWs in the North and Tropical Pacific. Future studies should investigate the spatial variability of the response in these two regions. The results in the North Pacific CO_2_ sink complete previous studies of ENSO-related PMHWs in the Tropical Pacific, and together with the new results obtained in this study thus provide a comprehensive view on the interplay between PMHWs and CO_2_ sensitive areas in the North and Tropical Pacific as illustrated in Fig. 3.

Over the 1985–2017 period, considering the duration and affected areas in the North Pacific and Tropical Pacific, we derive an integrated flux anomaly linked to PMHWs of 13 ± 6 TgC (Teragrams of Carbon = 10^12^ grams of Carbon) and −118 ± 25 TgC in the North Pacific and Tropical Pacific respectively. The climatological integrated fluxes expected during the same time and within the same area are −119 ± 12 TgC and 307 ± 31 TgC respectively. This corresponds to a flux anomaly of 11 ± 5% and 39 ± 11% for the respective regions. While the effect in absolute terms appears small at first sight, particularly in the North Pacific (only about 0.5% of all annual marine uptake of anthropogenic CO_2_^37^), increasing duration and intensity of the heat waves become relevant for closing the current carbon budget imbalance (currently in the order of ±100–500 TgC/yr for the past decades and recent years (see Table 6 in Friedlingstein et al.^37^) and should therefore receive attention in future budgets.

In this study, we have introduced the concept of intense and long-lasting MHWs, here defined as PMHWs, with a criterion based on the duration and the mean SST anomaly (i.e., duration > 38 days and mean SST anomaly > 2.3 degrees Celsius). Our results have shown that PMHWs occur predominantly in the major CO_2_ source and sink areas. Shorter-lasting MHW events are more widespread than PMHWs^10,19^ and further ensemble-based analyses are needed to understand on how their impact might affect air-sea CO_2_ exchanges at global scale. Except for one single product (JENA), all other observational-based products are available at monthly resolution which makes such investigation not possible. Future analyses based on an ensemble of CO_2_ products of high temporal resolution (i.e. < 30 day) would be needed to study the integrated impact of shorter extreme events on ocean CO_2_ fluxes.

In the North Atlantic and the mid-high latitude Southern Ocean CO_2_ sinks, no change is observed for the CO_2_ fluxes due to PMHWs, suggesting that the thermal effect and non-thermal effects cancel each other out. To further analyze this hypothesis, the use of reanalyzes is needed. Currently, long-term in-situ observations for pCO_2_, temperature, salinity, DIC and ALK are lacking in these areas preventing the establishment of robust air-sea CO_2_ flux products, climatologies and reanalysis skill validation for such a process study. Hence, PMHW-induced impacts on CO_2_ fluxes mechanisms in the Atlantic and mid-high latitude Southern Ocean remain unanswered, and increased monitoring efforts are needed accordingly.

**Fig. 4:**
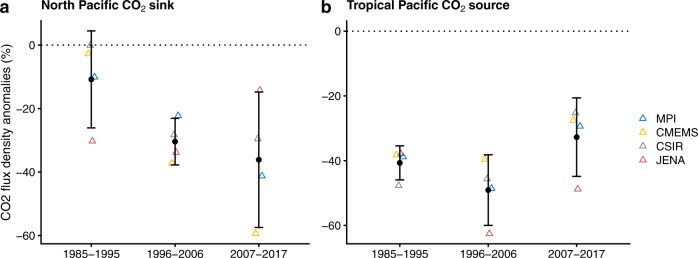
Evolution of F_CO2_ anomalies due to PMHWs during the 1985–2017 period. Trimmed average percent F_CO2_ anomalies during PMHWs for three time-periods (1985–1995, 1996–2006, 2007–2017) derived from an ensemble of 4 observation-based products of F_CO2_ (see section methods) in **a** North Pacific CO_2_ sink and **b** the Tropical Pacific CO_2_ source regions. The ensemble mean and standard deviation are given in black. An additional 12% uncertainty resulting from uncertain gas exchange^60^ has been added to the ensemble spread. The calculation of the percent F_CO2_ anomalies is detailed in the method section.

There has been a statistically significant increase (*P* = .08) in the attenuation in F_CO2_ due to PMHWs between the 1985–1995 and the 2007–2017 period in the North Pacific, and we have compared the distribution of the ensemble of the 4 observation-based products. On the contrary, there has been no statistically significant change in the Tropical Pacific (Fig. 4). Similarly, PMHWs have also increased in intensity in the North Pacific over the last decades (linear trend= 0.11 ± 0.03 °C/decade, *P* = .002), while their strength remains similar in the Tropics (linear trend= 0.04 ± 0.09 °C/decade, *P* = .66) (Fig. S4). Based on the results of our process study, we can develop the hypothesis that the reported increase in the intensity of PMHWs has potentially amplified the outgassing of CO_2_ over the 1985–2017 period, and that the competing mechanism, i.e., anomalous advection of DIC, was unable to counter-interact the thermal effect over this time scale. However, we cannot test such hypothesis as it would require a decomposition of F_CO2_ and DIC budgets from 1985 to 2017, the latter being currently not estimated by the reanalysis over this period. MHWs are projected to become stronger, more frequent and longer lasting in a warming climate^1,9,12,13^. Therefore, it is crucial to understand how PMHWs and F_CO2_ interact over longer time scales if we want to further unravel the evolution of the oceanic carbon cycle under climate change.

## Methods

### Observation-based products of F_CO2_

In this study, we use an ensemble of four observation-based products to quantify the impact of PMHWs on F_CO2_ in the three largest oceanic CO_2_ sink and the largest oceanic CO_2_ source in the Tropical Pacific from 1985 to 2017. Here, we provide a brief outline of the chosen products. More detail can be found in their respective publications.

The first observation-based product, from the Max Plank Institute for Meteorology (hereinafter denoted MPI)^47,48^, is based on a self-organizing map–feed-forward network that reconstructs the sea surface partial pressure of CO_2_ (spCO_2_) from various environmental predictor data. In a first step, the ocean is divided into biogeochemical regions of similar spCO_2_ properties (making use of a spCO_2_ climatology) and in a second step the non-linear relationship between auxiliary driver data and sparse observations is reconstructed to fill measurement gaps. The period of analysis is from 1982 to 2019 at monthly intervals and with a spatial resolution of 1° × 1°. It is based on a collection of ship and mooring spCO_2_ measurements assembled by the Surface Ocean CO_2_ Atlas (SOCAT) version 2020^49–52^.

The second observation-based product, from Copernicus Marine Environmental Monitoring Service (hereinafter denoted CMEMS)^36^, is from an ensemble-based forward feed neural network that reconstruct change in spCO_2_ from environmental predictor data. The period of analysis is from 1985 to 2018 at monthly intervals and with a spatial resolution of 1° × 1°. It is based on a collection of ship and mooring spCO_2_ measurements assembled by the Surface Ocean CO_2_ Atlas (SOCAT) version 2019^49–52^.

The third observation-based product, from the Council for Scientific and Industrial Research (hereinafter denoted CSIR)^53^, is from a machine-learning ensemble average of six two-step clustering-regression models that reconstruct change in spCO_2_ from environmental predictor data. The period of analysis is from 1982 to 2019 at monthly intervals and with a spatial resolution of 1° × 1°. It is based on a collection of ship and mooring spCO2 measurements assembled by the Surface Ocean CO_2_ Atlas (SOCAT) version 2019^49–52^.

The fourth observation-based product, from the Max Plank Institute for Biogeochemistry (hereinafter denoted JENA)^54^, is from an observation-driven ocean mixed-layer scheme that reconstruct change in spCO_2_ by fitting a data-driven diagnostic model of ocean mixed-layer biogeochemistry to surface-ocean CO_2_ partial pressure data from the SOCAT version 2019^49–52^. The period of analysis is from 1957 to 2019 at daily intervals and with a spatial resolution of 2.5° × 2°. The daily fields were averaged into monthly fields.

Finally, to evaluate the skill of the BGC reanalysis in estimating F_CO2_ anomalies associated with PMHWs in the North Pacific CO_2_ sink, we use an additional observation-based product, from the Japan Meteorology Agency (hereinafter denoted JMA)^55^, which is excluded from the spCO_2_ ensemble as its period of analysis is shorter than the previously listed products, i.e. from 1990 to 2018. This product is based on multiple linear regressions that reconstruct change in spCO_2_ from a set of environmental drivers. The temporal resolution is monthly intervals and with a spatial resolution of 1° × 1°. It is based on a collection of ship and mooring spCO_2_ measurements assembled by the Surface Ocean CO_2_ Atlas (SOCAT) version 2019^49–52^.

### Estimates of the air-to-sea flux density of CO_2_ from spCO_2_ data

In the five observation-based products and the BGC reanalysis, the air-to-sea CO_2_ flux density (F_CO2_) is generated from spCO_2_ data using the gas exchange formulation^56^,

1 $${{{{{{{{{{\rm{F}}}}}}}}}}}_{{{{{{{{{{\rm{CO}}}}}}}}}}2}={{{{{{{{{\rm{k}}}}}}}}}}{{{{{{{{{\rm{\alpha }}}}}}}}}}({{{{{{{{{{\rm{spCO}}}}}}}}}}}_{2}-{{{{{{{{{{\rm{pCO}}}}}}}}}}}_{2{{{{{{{{{\rm{atm}}}}}}}}}}}),$$

where α is the CO_2_ solubility in seawater, k, a gas transfer coefficient, pCO_2atm_ is the atmospheric partial pressure of CO_2_ and spCO_2_ is the sea surface partial pressure of CO_2_. Here, negative values of F_CO2_ indicate uptake of CO_2_ from the atmosphere to the ocean, while positive values indicate outgassing of CO_2_ from the ocean to the atmosphere. Each product performs their own calculation of the fluxes and the methods are described in the respective publications.

### Calculation of 2009–2017 monthly anomalies

In the reanalysis and the observation-based products, monthly anomalies (hereinafter denoted with a prime) are computed by removing a climatological value (hereinafter denoted with an overbar). The climatological value corresponds to the sum of a long-term linear trend and a monthly mean value. The monthly mean values are computed from the detrended monthly data.

### Calculation of 1985–2017 percent F_CO2_ anomalies

The percent F_CO2_ anomalies during PMHWs and for the 1985–2017 period correspond to the monthly F_CO2_ anomalies divided by the monthly F_CO2_ climatological values. The anomalies and climatological values were computed following the method detailed previously, with the exception that monthly mean values were only computed from the detrended monthly data over the 1985–1995 period. During this decade, the number of PMHWs per year, and near globally, were the lowest of the 1985–2017 period (see Fig. S5). By calculating the anomalies relative to this “reference” decade, we make sure that the percent F_CO2_ anomalies represent a change with respect to oceanic conditions not impacted by PMHWs.

In Fig. 1b, we represent, in each CO_2_ sink/source, an ensemble of four 1985–2017 trimmed mean percent F_CO2_ anomalies derived from the observation-based products. We use the trimmed mean instead of the mean because it is a robust estimator of central tendency and provides a better estimation of the location of the bulk of the data than the mean when the distribution is asymmetric, which is the case here. More precisely, we use a 5% trimmed mean, i.e., the lowest 5% and the highest 5% of the data are excluded.

### Taylor expansion of F_CO2_ anomalies

To determine the driving mechanisms causing F_CO2_ anomalies during PMHWs in the North Pacific CO_2_ sink, we calculate a first-order Taylor series expansion of F_CO2_ anomalies in terms of its driving parameters (i.e., wind, upper ocean temperature, salinity, dissolved inorganic carbon (DIC), alkalinity (ALK) and the atmospheric partial pressure of CO_2_)^28,44^.

First, we performed the linear Taylor decomposition of Eq. (1):

2 $${F}_{{{CO}_{2}}^{\prime}} \,\approx\, \left(k\alpha \right)^{\prime} \overline{\left({{spCO}}_{2}-{{pCO}}_{2{atm}}\right)}+\,\overline{\left(k\alpha \right)} {{{spCO}}_{2}}^{\prime} -\overline{\left(k\alpha \right)} {{{pCO}}_{2{atm}}}^{\prime}.$$

The right-hand-side terms represent the contribution to F_CO2_’ of gas transfer and solubility anomalies, atmospheric pCO_2_ anomalies and spCO_2_ anomalies. Note that the temperature dependence of *k* and *α* cancel each other, and (*kα*)′ is mainly driven by variations in wind speed^28,44^.

The spCO_2_ anomalies are further decomposed into contributions from sea surface temperature anomalies (SST’), sea surface dissolved inorganic carbon anomalies (SDIC’), sea surface alkalinity anomalies (SALK’) and sea surface salinity anomalies (SSS’), neglecting the second-order terms^28,44,45,57^:

3 $${{{spCO}}_{2}}^{\prime} \approx \frac{\partial {{spCO}}_{2}}{\partial {SDIC}}{SDIC}^{\prime}+\frac{\partial {{spCO}}_{2}}{\partial {SALK}}{SALK}^{\prime}+\frac{\partial {{spCO}}_{2}}{\partial {SST}}{SST}^{\prime}+\frac{\partial {{spCO}}_{2}}{\partial {SSS}}{SSS}^{\prime} .$$

Substituting Eq. (3) into Eq. (2) gives the contributions of all parameters to F_CO2_’ in a single expression:

4 $${{F}_{{CO}2}}^{\prime} \,\approx \,	 \left(k\alpha \right)^{\prime} \overline{\left({{spCO}}_{2}-{{pCO}}_{2{atm}}\right)} \\ 	+\,{\overline{\left(k\alpha \right)}}\,\left(\frac{\partial {{spCO}}_{2}}{\partial {SSDIC}}{SDIC}^{\prime}+\frac{\partial {{spCO}}_{2}}{\partial {SSALK}}{SALK}^{\prime}+\frac{\partial {{spCO}}_{2}}{\partial {SST}}{SST}^{\prime}+\frac{\partial {{spCO}}_{2}}{\partial {SSS}}{SSS}^{\prime} \right)\\ 	-\,\overline{\left(k\alpha \right)}{{{pCO}}_{2atm}}^{\prime} .$$

Here, the sea surface quantities correspond to the quantities at the first level of the ocean reanalysis estimates (z ~ −0.50 m). Following Doney et al.^28^, the partial derivatives in Eq. (4) were computed off-line at each grid point, taking SDIC as an example, as:

5 $$\frac{\partial {{spCO}}_{2}}{\partial {SDIC}}\approx \frac{{{spCO}}_{2}\left({SDIC},\overline{{SAlk}},\overline{{SST}},\overline{{SSS}}\right)-{{spCO}}_{2}\left(\overline{{SDIC}},\overline{{SALK}},\overline{{SST}},\overline{{SSS}}\right)}{{SDI}{C}^{{\prime} }}.$$

where spCO2 values are calculated using the seacarb program for R (https://CRAN.R-project.org/package=seacarb).

### DIC anomalies budget

In our study, we show that DIC anomalies play a significant role in controlling F_CO2_ anomalies during PMHWs. We therefore conduct a DIC anomalies budget to elucidate what processes controlled DIC anomalies.

In the ocean reanalysis, the changes in DIC concentration with time are described by the following equation:

6 $$\frac{\partial {DIC}}{\partial t}={{ADV}}_{H}+{{ADV}}_{z}+{{DIFF}}_{z}+{SBC}+{F}_{{CO}2}+B+r$$

where *ADV*_*H*_ and *ADV*_*_z*_ are the horizontal and vertical advection of DIC respectively, *DIFF*_*z*_ is the vertical diffusion of DIC, *SBC* are the freshwater fluxes that dilute or concentrate DIC, *F*_*CO*2_ is the air-sea CO_2_ flux density, *B* is the biological activity that consumes or releases DIC (see details in Aumont et al.^58^), and *r* is the climatological damping (see supplementary information). Positive values result in a net increase in DIC. All terms were computed online on a daily basis and stored for monthly averages. The DIC tendency (rate of change or trend) equation (Eq. 6) is expressed as a function of monthly anomalies and averaged over the average mixing layer observed during PMHWs in the reanalysis (indicated by angle brackets), i.e. from the surface to h ~ 47 m:

7 $$\frac{\partial < {DIC}^{\prime} > }{\partial t}= < {{{ADV}}_{H}}^{\prime} > \,+{ < {{ADV}}_{z}}^{\prime} > \,+\, < {{{DIFF}}_{z}}^{\prime} > \,+\, < {SBC}^{\prime} > \,+\frac{ < {{F}_{{CO}2}}^{\prime} > }{h}+\, < B^{\prime} > \,+ < r^{\prime} > .$$

### Satellite sea surface temperature and marine heatwaves detection

MHWs locations, dates of onset and durations were derived from the global daily remotely sensed National Ocean Atmospheric Administration (NOAA) Optimum Interpolation sea surface temperature V2, ¼° gridded data over 1982–2017^33,34^. This dataset is derived from the advanced very high-resolution radiometer (AVHRR).

We apply a standard MHW detection algorithm^2^ to the gridded SST data. More specifically, a warm event is considered as a MHW if it lasts for 5 or more days, with sea surface temperatures warmer than the 90th percentile based on a 1983–2012 historical climatology. The MHW detection algorithm is usually not performed on grid cells with periods of ice coverage longer than 5 days^10^. We therefore restrict our analysis to the area between 60°S and 60°N. For each MHW detected, the date of onset, duration and mean sea surface temperature anomaly are estimated by the MHW detection algorithm.

### Calculation of anomalies associated with PMHWs

For each PMWH detected, the monthly anomalies were extracted at the model or observation-based products grid-point the closest to the PMHW location and for the entire duration of the PMHW. Then, to match the temporal resolution of the PMHW, the extracted anomalies were resampled from monthly to daily frequency through linear interpolation. The interpolated values were then averaged over the duration of the PMHW to give a single value, consistently with the other metrics derived from the MHW detection algorithm.

## Supplementary information

Supplementary Information

## Data Availability

The reanalysis data can be downloaded from the Copernicus Marine Environmental Monitoring Service (https://resources.marine.copernicus.eu/product-detail/GLOBAL_ANALYSIS_FORECAST_BIO_001_028/INFORMATION). The DIC budget terms data are available upon request from the corresponding author. The BGC-Argo data were downloaded from the Argo Global Data Assembly Centre in France (ftp://ftp.ifremer.fr/argo/). The observation-based product are available from the Surface Ocean pCO2 Mapping Intercomparison website (http://www.bgc-jena.mpg.de/SOCOM/). The SST data are provided by NOAA/ESR/PSL at https://psl.noaa.gov/data/gridded/data.noaa.oisst.v2.highres.html.
