# Supplementary material for: Decrease in air-sea CO2 fluxes caused by persistent marine heatwaves
Source: Nat Commun. 2022 Jul 25;13:4300. doi: 10.1038/s41467-022-31983-0 (PMC9314444; doi:10.1038/s41467-022-31983-0)
Supplement: Supplementary file 1 — Supplementary Information [file 41467_2022_31983_MOESM1_ESM.pdf]

Supplementary Note 1: Biogeochemical ocean reanalysis

To understand the interaction between persistent marine heatwaves (PMHWs) and the air-sea CO_2 flux density (F_{CO2}) in the North Pacific CO_2 sink, we use a biogeochemical reanalysis of the global ocean based on a Global Ocean hydrodynamic-biogeochemical model, implemented and operated by the Copernicus Marine Environment Monitoring Service (CMEMS) Global Monitoring and Forecasting Center[1]. It features the offline coupled NEMO–PISCES model[2], with a 1/4° horizontal resolution, daily temporal resolution from 2009 to 2017 and 50 vertical levels (with 22 levels in the upper 100 m, the vertical resolution is 1 m near the surface and decreases to 450 m resolution near the bottom). The daily fields were averaged into monthly fields.

The biogeochemical model PISCES v2[2] features 24 prognostic variables and includes five nutrients that limit phytoplankton growth (nitrate, ammonium, phosphate, silicate and iron) and four living compartments: two phytoplankton groups (nanophytoplankton and diatoms) and two zooplankton size classes (microzooplankton and mesozooplankton, resp. small and large); the bacterial pool is not explicitly modelled. PISCES distinguishes three non-living pools for organic carbon (semi-labile dissolved organic carbon, small sinking particles, and large sinking particles), particles of calcium carbonate and biogenic silicate. Additionally, the model simulates the carbonate system and dissolved oxygen. PISCES has been successfully used in a variety of biogeochemical studies, both at regional and global scale [3–10].

The dynamical component is the latest Mercator Ocean global 1/12° high-resolution ocean model system, extensively described and validated in Lellouche et al.[11,12]. This system provides daily and 1/4°-coarsened fields of horizontal and vertical current velocities, vertical eddy diffusivity, mixed layer depth, sea ice fraction, potential temperature, salinity, sea surface height, surface wind speed and net surface solar shortwave irradiance that drive the transport of biogeochemical tracers. This system also features a reduced-order Kalman filter based on the Singular Evolutive Extended Kalman filter (SEEK) formulation introduced by Pham et al. [13], that assimilates, on a 7-day assimilation cycle, along-track altimeter data, satellite Sea

Surface Temperature and Sea-Ice Concentration from OSTIA[14], and *in situ* temperature and salinity vertical profiles from the CORA 4.2 *in situ* database[15].

The biogeochemical component of the coupled system also embeds a reduced order Kalman filter (similar to the above mentioned) that operationally assimilates daily L4 remotely sensed surface chlorophyll. In parallel, a climatological-damping is applied to nitrate, phosphate, oxygen, silicate - with World Ocean Atlas 2013 - to dissolved inorganic carbon and alkalinity - GLODAPv2 climatology[16]- and to dissolved organic carbon and iron - with a 4000-year PISCES climatological run. This relaxation is set to mitigate the impact of the physical data assimilation in the offline coupled hydrodynamic-biogeochemical system, engendering significant rises of nutrients in the Equatorial Belt area, and resulting in an unrealistic drift of various biogeochemical variables e.g. chlorophyll, nitrate, phosphate[17,18]. The time-scale associated with this climatological damping is set to 1 year and allows a smooth constraint to reduce the model drift.

Supplementary Note 2. Comparison with observation-based products of F_{CO2}

We evaluate the skill of the biogeochemical (BGC) reanalysis through the estimation of F_{CO2} anomalies associated with PMHWs in the North Pacific against an ensemble of 5 observation-based products of F_{CO2} (see method section). The period of analysis is from 2009 to 2017 and has been chosen to encompass the period coverage of the six datasets. All products estimate positive F_{CO2} anomalies during PMHWs in the North Pacific CO_2 sink (Figure S1). The average reduction in F_{CO2} during PMHWs ranges from 0.011+/-0.10 molC/m2/year (mean +/- 95% confidence interval) to 0.352 +/-0.10 molC/m2/year (the reanalysis). The difference between the reanalysis and the observation-based products is within the same order of magnitude than the observation-based products inter-difference. This in turn provides good confidence that the reanalysis is as skilful as the observation-based products to estimate F_{CO2} anomalies due to PMHWs in the North Pacific CO_2 sink.

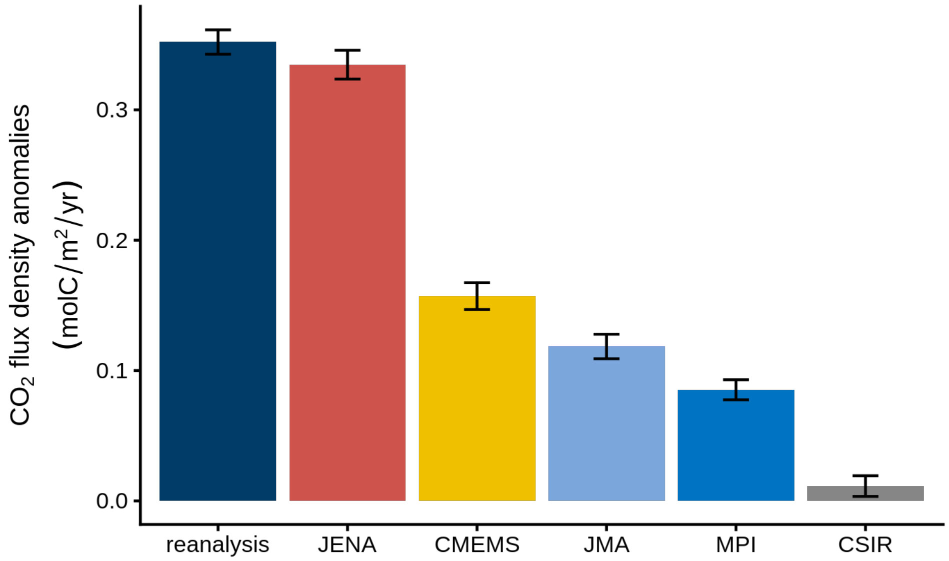

Figure S1. Average F_{CO2} anomalies during PMHWs in the North Pacific CO_2 sink estimated using the BGC reanalysis and an ensemble of 5 observation-based products of F_{CO2} for the 2009-2017 period. The calculation is performed on the SST grid points that have experienced at least 3 PMHWS from 1985 to 2017, as explained in the main text. The error bars correspond to 95 % confidence intervals. An additional 12 % uncertainty resulting from uncertain gas exchange [19] has been added to the 95 % confidence interval of the observations-based products.

Supplementary Note 3. Comparison with BGC-Argo floats observations

We further test the skill of the BGC reanalysis in reproducing anomalies in the four oceanic drivers known to control F_{CO2} (temperature, salinity, dissolved inorganic carbon, (DIC) and alkalinity, (ALK)) during PMHWs through a comparison to observations from 6 BGC-Argo floats that profiled north of the ocean region impacted by the 2013/2015 'warm blob' PMHW (Fig. S2) [20,21].

DIC and ALK are not measured by BGC-Argo floats, but they are well estimated (with a mean uncertainty of 7.1 μmol/kg and 6.3 μmol/kg respectively) from the neural network CANYON-B [22] coupled with BGC-Argo floats "Delayed Mode" measurements of floats pressure, temperature, salinity and oxygen associated with the geolocation and date of sampling ("Delayed Mode" is referring to the highest quality of data possible in the Argo data system)[23]. We used the observations from 6 BGC-Argo floats that sampled the Gulf of Alaska in a region of similar water mass from January 2009 to December 2017. The float data were downloaded from the Argo Global Data Assembly Centre in France (ftp://ftp.ifremer.fr/argo/). The CTD and trajectory data were quality controlled using the standard Argo protocol[24], and the oxygen measurements according to Thierry et al. [25,26].

The monthly reanalysis outputs were collocated in time and the closest to the BGC-Argo profiles positions. The matchups were generated by interpolating the model data to the sampling pressure of the float data. The reanalysis oxygen data were transformed from mmol m^{-3} into μmol kg^{-1} (native units of BGC-Argo oxygen measurements) using the reanalysis temperature and salinity. The monthly anomalies were computed as follow. First, vertical profiles of temperature, salinity, DIC and ALK collected with the BGC-Argo floats and estimated with the ocean reanalysis were depth-averaged from the surface to z= - 47 m; the latter corresponding to the average mixing layer observed during PMHWs in the reanalysis. Then, the anomalies were constructed by removing a monthly mean climatology computed from 2009 to 2017. Finally, the anomalies were monthly-averaged.

The region sampled by the float was not impacted by PMHWs as per our definition (mean surface temperature greater than 2.3 °C and for a period longer than 38 days), it was nevertheless subject to an important increase in sea surface temperature for an extended period of time (~1.5°C for almost 2 years). The ocean region impacted by PMHWs is located

roughly 1660 km south of the floats location, in the 'warm blob region' defined by Bond et al. [27](black box in Fig. S2a).

The time series of temperature and salinity anomalies estimated by the ocean reanalysis compare well to the float observations with a mean bias of 0.04 °C and -0.001 psu respectively from November 2013 to October 2015 (Fig. S2c). The timing and the intensity of the negative DIC and ALK anomalies are correctly represented by the reanalysis. The reanalysis tends to slightly underestimate the negative DIC and ALK anomalies, with a small negative of bias of 2.2 μmol/kg and -0.9 μmol/kg, which however remains within the uncertainties of the observed DIC and ALK, i.e., 7.1 μmol/kg and 6.3 μmol/kg [22].

Moreover, consistent with our results from all PMHWs in the North Pacific CO_2 sink (Figure S1), there is a positive F_{CO2} anomaly (Figure S2a) from 2014 to 2015 in the 'warm blob' region. PMHWs were the most abundant in this region from November 2013 to October 2015 (Figure S2b). Thus, these results demonstrate that the reanalysis is skilled to represent the ocean processes that lead to positive F_{CO2} anomaly during the 'warm blob' and hence during PMHWs in the North Pacific.

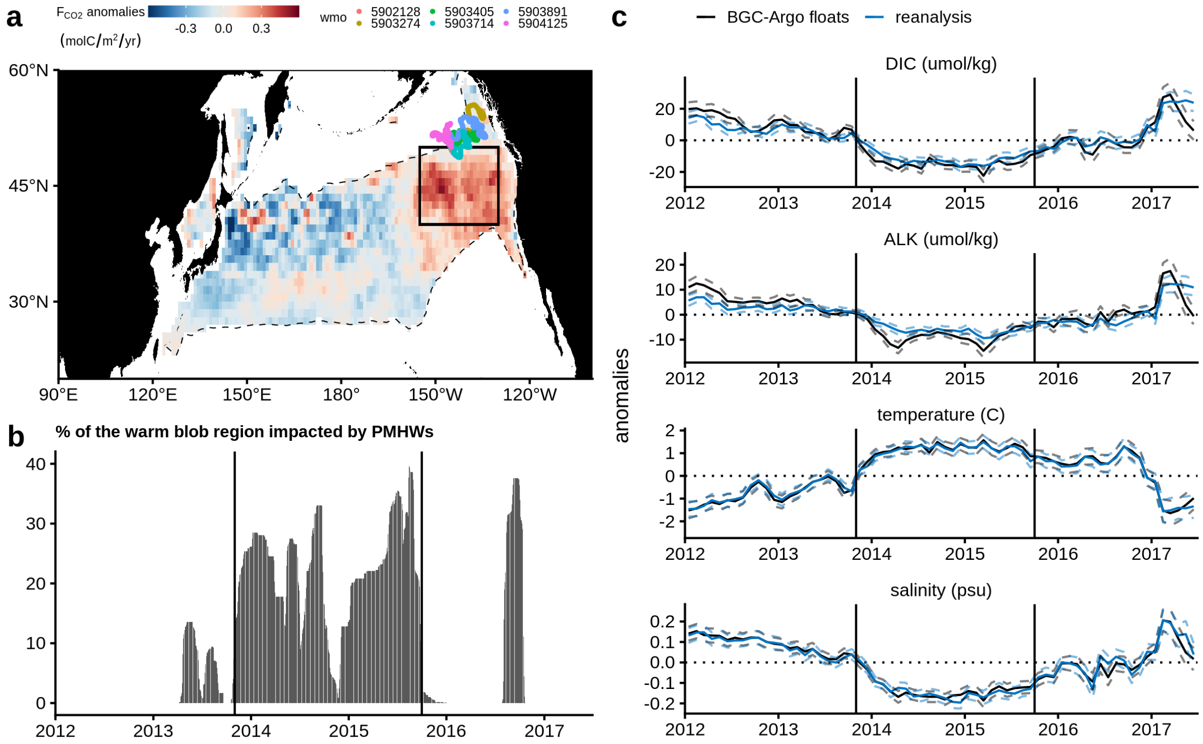

Figure S2 . (a) Mean 2014-2015 F_{CO2} anomalies derived from the ocean reanalysis. The black box (-155 - -135°W, 40 - 50 °N) indicates the 'warm blob' region as defined in Bond et al.[27]. The positions of the 6 BGC-Argo floats from 2009 to 2017 used to assess the quality of the ocean reanalysis are also indicated (colored dots). The numbers represent the World Meteorological Organization number of the BGC-Argo floats. **(b)** Percentage of the 'warm blob' region impacted by PHMWS each day. **(c)** Time series of monthly 0-50 m depth-averaged DIC, ALK, temperature and salinity anomalies estimated from the BGC-Argo floats observations (black lines) and from the ocean reanalysis at the floats' locations (blue lines). Dashed lines represent standard error of the monthly mean climatologies. The vertical lines in **(b)** and **(c)** represent the period during which PMHWs were the most abundant in the 'warm blob' region, i.e. from November 2013 to October 2015.

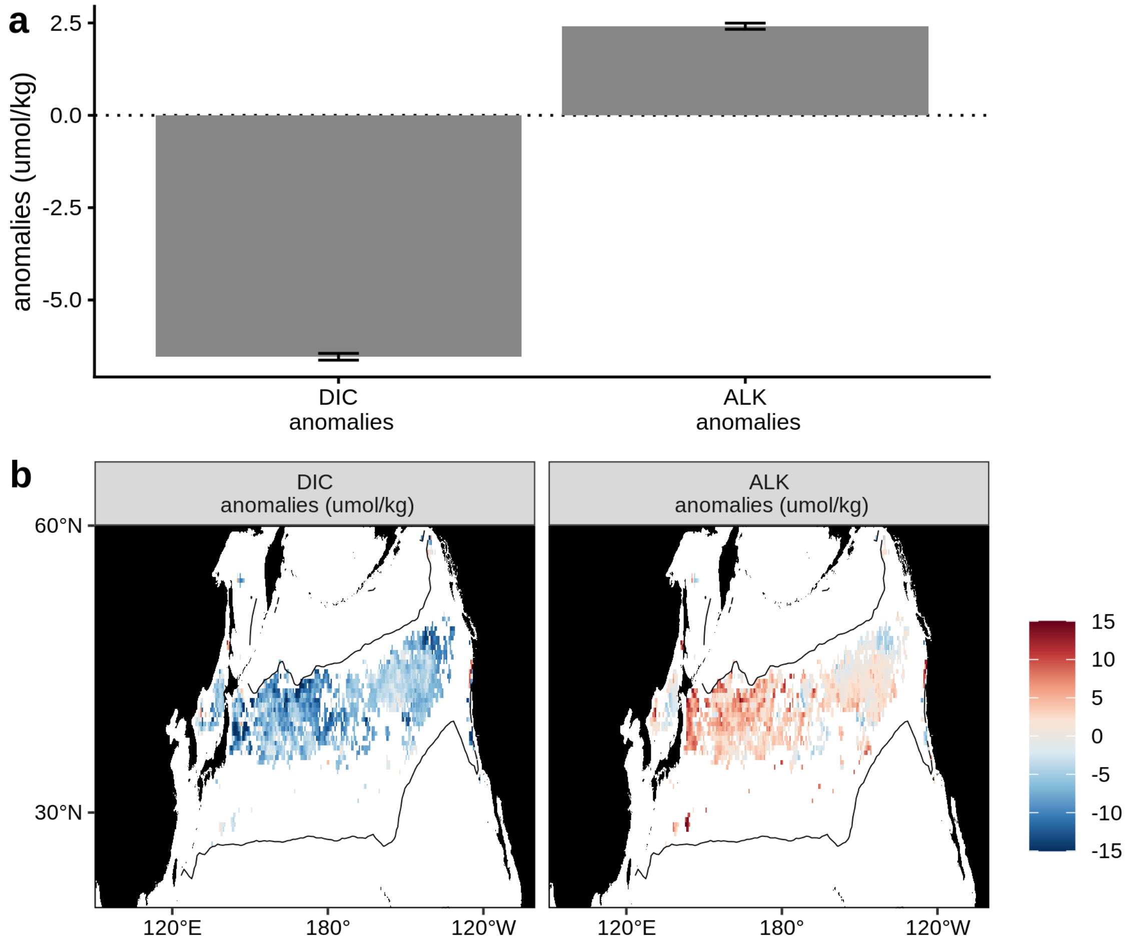

Figure S3. (a) Average dissolved inorganic carbon (DIC) and alkalinity (ALK) anomalies at the first level of the ocean reanalysis (z ~ - 0.50 m) during PMHWs in the North Pacific CO_2 sink derived from the BGC reanalysis for the 2009-2017 period. The calculation is performed on the SST grid points that have experimented at least 3 PMHWS from 1985 to 2017, as explained in the main text. The error bars correspond to 95 % confidence intervals. The panel **(b)** represents the spatial distribution of the DIC and ALK anomalies for the 2009-2017 period. Note that the spatial repartition of ALK anomalies is somewhat more heterogeneous than for DIC anomalies. Even though, the average over the entire domain is positive, there are few local places where ALK anomalies are negative, as for example in the North-eastern part of the basin. This explain why the ALK anomalies observed by the BGC-Argo floats are negative (Figure S2c). However, given the small contribution of ALK anomalies to F_{CO2} anomalies during PMHWs in the North Pacific CO_2 sink, this small heterogeneity is inconsequential for the conclusions of the study.

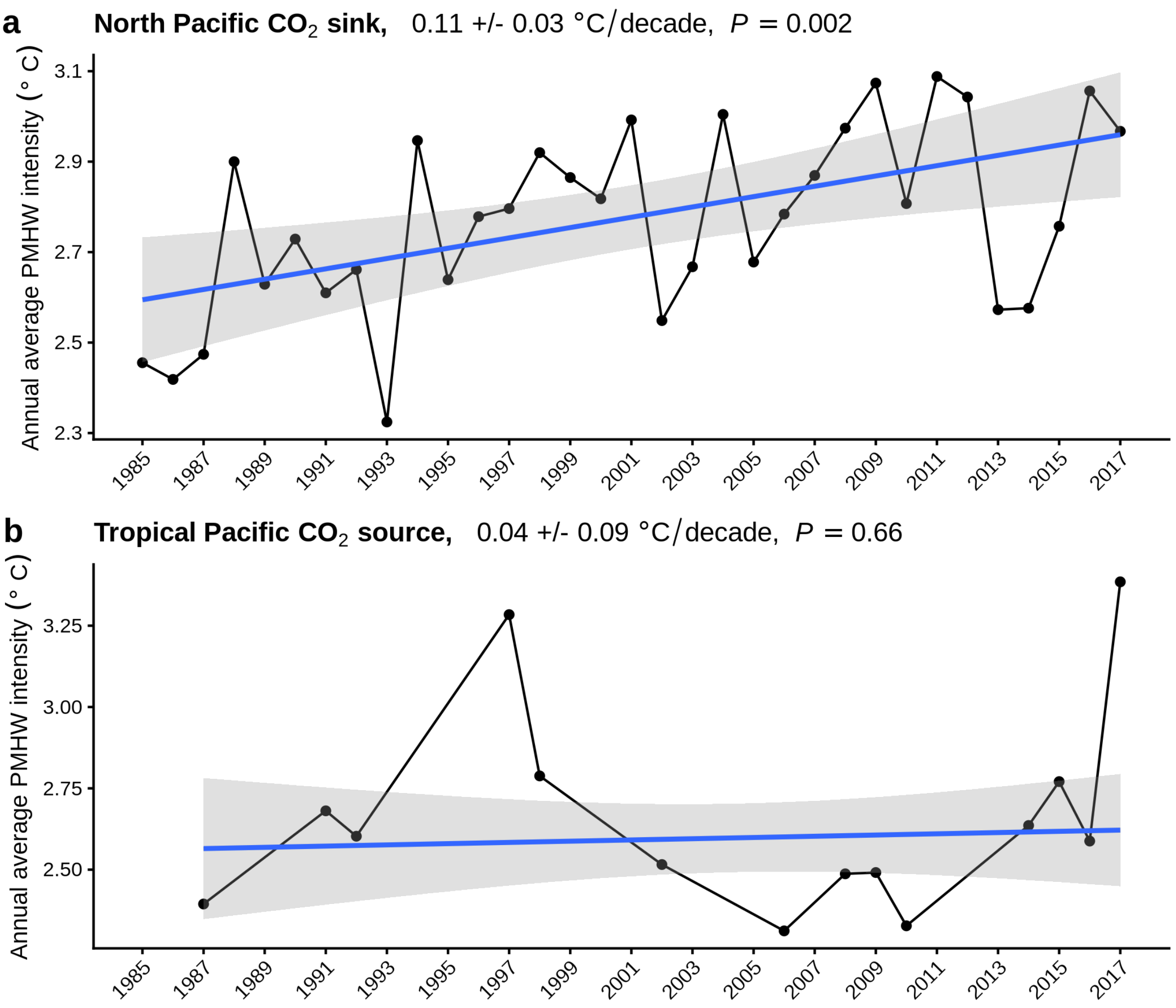

Figure S4. Annual average PMHWs intensity in the North Pacific CO_2 sink, **(a)**, and in the Tropical Pacific CO_2 source, **(b)**, from 1985 to 2017. The calculation is performed on the SST grid points that have experimented at least 3 PMHWS from 1985 to 2017, as explained in the main text. The annual average PMHW intensity corresponds to PMHW intensities (i.e., mean sea surface temperature anomalies averaged over the PMHW duration) averaged over all PMHWs that occur for a given year from 1985 to 2017. The blue line and the gray shading represent the linear trend and its 95 % confidence interval.

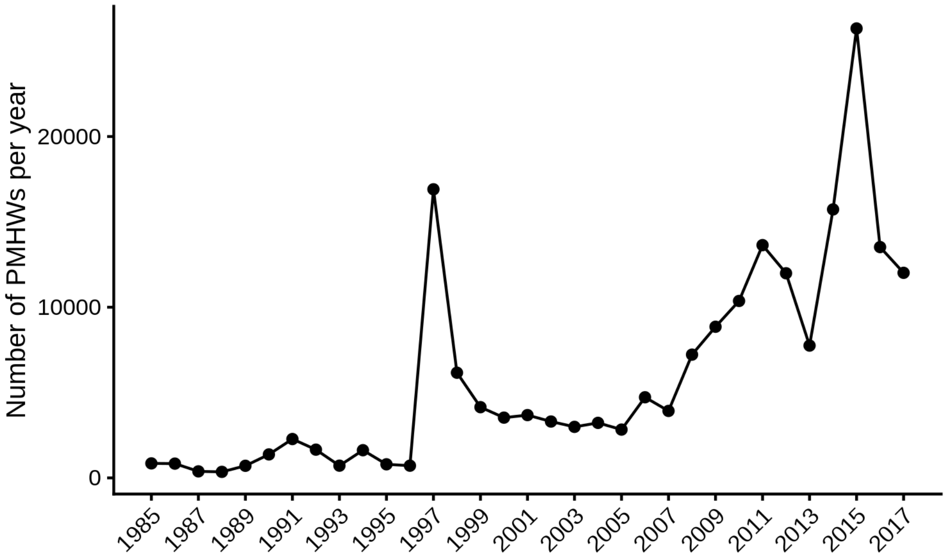

Figure S5. Number of PMHWs per year, and near-globally, i.e. between 60°S and 60°N, from 1985 to 2017.

Table S1. Results of the Yuen's trimmed mean test that assesses the significance of the trimmed average percent F_{CO2} anomalies reported in Figure 1b.

critical CO_2 sink/source regions	Yuen's trimmed mean test	Observational-based products			
		CMEMS	CSIR	JENA	MPI
Tropical Pacific CO_2 source	Trimmed average	-33.9	-36.7	-52.2	-38.4
	95 % confidence interval	-34.4 / -33.4	-37.3 / -36.2	-52.8 / -51.7	-38.9 / -37.8
	P	< .001	< .001	< .001	< .001
North Pacific CO_2 sink	Trimmed average	-42.5	-23.6	-21.3	-30.2
	95 % confidence interval	-44.0 / -41.0	-24.4 / -22.7	-22.9 / -19.7	-31.3 / -29.0
	P	< .001	< .001	< .001	< .001
North Atlantic CO_2 sink	Trimmed average	-5.2	- 5.4	2.0	-7.4
	95 % confidence interval	-6.2 / -4.1	-6.7 / -4.1	0.9 / 3.1	-8.5 / -6.3
	P	< .001	< .001	< .001	< .001
Mid-high latitude southern oceans CO_2 sink	Trimmed average	4.7	6.2	-4.2	1.2
	95 % confidence interval	4.3 / 5.1	5.9 / 6.5	-4.5/ -3.9	0.9/ 1.4
	P	< .001	< .001	< .001	< .001

Supplementary References

1. Le Traon, P. Y. *et al.* From Observation to Information and Users: The Copernicus Marine Service Perspective. *Front. Mar. Sci.* **6**, 234 (2019).
2. Aumont, O., Ethé, C., Tagliabue, A., Bopp, L. & Gehlen, M. PISCES-v2: an ocean biogeochemical model for carbon and ecosystem studies. *Geosci. Model Dev.* **8**, 2465–2513 (2015).
3. Bopp, L., Aumont, O., Cadule, P., Alvain, S. & Gehlen, M. Response of diatoms distribution to global warming and potential implications: A global model study. *Geophys. Res. Lett.* **32**, (2005).
4. Gehlen, M. *et al.* Reconciling surface ocean productivity, export fluxes and sediment composition in a global biogeochemical ocean model. *Biogeosciences* **3**, 521–537 (2006).
5. Gutknecht, E., Reffray, G., Mignot, A., Dabrowski, T. & Sotillo, M. G. Modelling the marine ecosystem of Iberia-Biscay-Ireland (IBI) European waters for CMEMS operational applications. *Ocean Sci.* **15**, 1489–1516 (2019).
6. Lefèvre, N. *et al.* Basin-Scale Estimate of the Sea-Air CO_2 Flux During the 2010 Warm Event in the Tropical North Atlantic. *J. Geophys. Res. Biogeosciences* **124**, 973–986 (2019).
7. Schneider, B. *et al.* Climate-induced interannual variability of marine primary and export production in three global coupled climate carbon cycle models. *Biogeosciences* **5**, 597–614 (2008).
8. Séférian, R. *et al.* Skill assessment of three earth system models with common marine biogeochemistry. *Clim. Dyn.* **40**, 2549–2573 (2013).
9. Steinacher, M. *et al.* Projected 21st century decrease in marine productivity: a multi-model analysis. *Biogeosciences* **7**, 979–1005 (2010).

10. Tagliabue, A. *et al.* Hydrothermal contribution to the oceanic dissolved iron inventory. *Nat. Geosci.* **3**, 252–256 (2010).

11. Lellouche, J.-M. *et al.* Evaluation of global monitoring and forecasting systems at Mercator Océan. *Ocean Sci.* **9**, 57–81 (2013).

12. Lellouche *et al.* Recent updates to the Copernicus Marine Service global ocean monitoring and forecasting real-time 1/12° high-resolution system. *Ocean Sci.* **14**, 1093–1126 (2018).

13. Tuan Pham, D., Verron, J. & Christine Roubaud, M. A singular evolutive extended Kalman filter for data assimilation in oceanography. *J. Mar. Syst.* **16**, 323–340 (1998).

14. Good, S. *et al.* The Current Configuration of the OSTIA System for Operational Production of Foundation Sea Surface Temperature and Ice Concentration Analyses. *Remote Sens.* **12**, 720 (2020).

15. CMEMS. https://resources.marine.copernicus.eu/?option=com_csw&view=details&product_id=INSITU_GLO_NRT_OBSERVATIONS_013_030.

16. Key, R. M. *et al. Global Ocean Data Analysis Project, Version 2 (GLODAPv2).* (Carbon Dioxide Information Analysis Center, Oak Ridge Nat Lab, 2015).

17. Fennel, K. *et al.* Advancing Marine Biogeochemical and Ecosystem Reanalyses and Forecasts as Tools for Monitoring and Managing Ecosystem Health. *Front. Mar. Sci.* **6**, 89 (2019).

18. Park, J.-Y. *et al.* Modeling Global Ocean Biogeochemistry With Physical Data Assimilation: A Pragmatic Solution to the Equatorial Instability. *J. Adv. Model. Earth Syst.* **10**, 891–906 (2018).

19. Roobaert, A., Laruelle, G. G., Landschützer, P. & Regnier, P. Uncertainty in the global oceanic CO2 uptake induced by wind forcing: quantification and spatial analysis. *Biogeosciences* **15**, 1701–1720 (2018).

20. Bif, M. B., Siqueira, L. & Hansell, D. A. Warm Events Induce Loss of Resilience in Organic Carbon Production in the Northeast Pacific Ocean. *Glob. Biogeochem. Cycles* **33**, 1174–1186 (2019).

21. Plant, J. N. *et al.* Net community production at Ocean Station Papa observed with nitrate and oxygen sensors on profiling floats. *Glob. Biogeochem. Cycles* **30**, 859–879 (2016).

22. Bittig, H. C. *et al.* An alternative to static climatologies: robust estimation of open ocean CO2 variables and nutrient concentrations from T, S, and O2 data using Bayesian neural networks. *Front. Mar. Sci.* **5**, 328 (2018).

23. Bittig, H. C. *et al.* A BGC-Argo guide: Planning, deployment, data handling and usage. *Front. Mar. Sci.* **6**, 502 (2019).

24. Wong, Keeley, Robert, Carval, Thierry, & Argo Data Management Team,. Argo Quality Control Manual for CTD and Trajectory Data. (2015) doi:10.13155/33951.

25. Thierry, V. *et al. Processing Argo oxygen data at the DAC level*. (Ifremer, 2018). doi:10.13155/39795.

26. Thierry, V. & Bittig, H. Argo quality control manual for dissolved oxygen concentration. (2018).

27. Bond, N. A., Cronin, M. F., Freeland, H. & Mantua, N. Causes and impacts of the 2014 warm anomaly in the NE Pacific. *Geophys. Res. Lett.* **42**, 3414–3420 (2015).
